# 

**Published:** 1890-06

**Authors:** 


CONTENTS:

PAGE

ORIGINAL ARTICLES:

Tuberculosis in Birds. By Walter K. Sibley, B.A., M.B., B.C., Cambridge; M.R.C.S., England . . . 317
The Horse's Hoof. By Dr. Williamson Bryden . . . 334
Age of the Horse, Ox, Dog, and other Domesticated Animals. By R. S. Huidekoper, M.D., Veterinarian . . . 340
Pig Feeding . . . 346

EDITORIAL DEPARTMENT:

United States Veterinary Medical Association . . . 347
Molar Extractor and Cutter . . . 350

CASES, SELECTIONS, TRANSLATIONS:

A Contribution to the Study of Parturient Fever of the Cow. By Professor Thomassen, Utrecht, Holland . . . 351

SOCIETY PROCEEDINGS:

The Keystone Veterinary Medical Association . . . 362
Long Island Veterinary Society . . . 364

VETERINARY COLLEGE NOTES:

Veterinary Department, University of Pennsylvania . . . 365

BOOKS AND PAMPHLETS RECEIVED . . . 365

